# Novel and recurrent variants of *ATP2C1* identified in patients with Hailey-Hailey disease

**DOI:** 10.1007/s13353-020-00538-8

**Published:** 2020-01-25

**Authors:** J. Sawicka, A. Kutkowska-Kaźmierczak, K. Woźniak, A. Tysarowski, K. Osipowicz, J. Poznański, A. M. Rygiel, N. Braun-Walicka, K. Niepokój, J. Bal, C. Kowalewski, K. Wertheim-Tysarowska

**Affiliations:** 1 Medical Genetics Department, Institute of Mother and Child, Kasprzaka 17a, 01-211 Warsaw, PL Poland; 2 Department of Dermatology and Immunodermatology, Medical University of Warsaw, Koszykowa 82A, 00-001 Warsaw, PL Poland; 3 Translational and Molecular Oncology Department, Maria Sklodowska-Curie Memorial Cancer Center and Institute of Oncology, W. K. Roentgena 5, 02-781 Warsaw, PL Poland; 4 Institute of Biochemistry and Biophysics, Polish Academy of Sciences, Pawińskiego 5A, 02-106 Warsaw, PL Poland

**Keywords:** Hailey-Hailey disease, *ATP2C1*, Genodermatosis

## Abstract

Hailey-Hailey disease (HHD) is a rare, late-onset autosomal dominant genodermatosis characterized by blisters, vesicular lesions, crusted erosions, and erythematous scaly plaques predominantly in intertriginous regions. HHD is caused by *ATP2C1* mutations. About 180 distinct mutations have been identified so far; however, data of only few cases from Central Europe are available. The aim was to analyze the *ATP2C1* gene in a cohort of Polish HHD patients. A group of 18 patients was enrolled in the study based on specific clinical symptoms. Mutations were detected using Sanger or next generation sequencing. In silico analysis was performed by prediction algorisms and dynamic structural modeling. In two cases, mRNA analysis was performed to confirm aberrant splicing. We detected 13 different mutations, including 8 novel, 2 recurrent (p.Gly850Ter and c.325-3 T > G), and 6 sporadic (c.423-1G > T, c.899 + 1G > A, p.Leu539Pro, p.Thr808TyrfsTer16, p.Gln855Arg and a complex allele: c.[1610C > G;1741 + 3A > G]). In silico analysis shows that all novel missense variants are pathogenic or likely pathogenic. We confirmed pathogenic status for two novel variants c.325-3 T > G and c.[1610C > G;1741 + 3A > G] by mRNA analysis. Our results broaden the knowledge about genetic heterogeneity in Central European patients with *ATP2C1* mutations and also give further evidence that careful and multifactorial evaluation of variant pathogenicity status is essential.

## Introduction

Hailey-Hailey disease (HHD, OMIM 16960, or Benign Chronic Pemphigus.) is a rare (incidence 1:50000) autosomal dominant genodermatosis. The symptoms, aggravating periodically, onset in third–fourth decade include blisters, vesicular lesions, crusted erosions, and erythematous scaly plaques, which occur mainly on groins, axillae, neck, and other intertriginous areas, and mucosa may also be involved. Lesions may be odorous and painful and lead to mobility affecting fissures (Li et al. 2016; Zamiri and Munro 2016). In histopathological findings, suprabasalar and intraepidermal keratinocyte acantholysis with a “dilapidated brick wall” appearance is due to abnormal epidermal Ca^2+^ distribution by secretory pathway Ca(2+) ATPase 1 (hSPCA1) caused by mutation in its gene: calcium-transporting ATPase type 2C member 1 (*ATP2C1*) (Cheng et al. 2010; Micaroni et al. 2016; Cialfi et al. 2016). Importantly, *ATP2C1* is expressed in all tissues, although HHD clinical symptoms are solely isolated to the skin. Four isoforms differing by alternative processing of the C-terminus are produced, but only few of *ATP2C1* mutations localized beyond the core of 26 exons are present in each transcript (Nellen et al. 2017). The majority of *ATP2C1* mutations lead to a premature termination codon (PTC); thus the dominant inheritance pattern of HHD seems to result from haploinsufficiency. Nevertheless, as around 1/3 of mutations lead to missenses or in-frame rearrangements, other mechanisms may be involved (Dobson-Stone et al. 2002; Kitajima 2002). Thus, to understand the pathophysiological molecular mechanism of HHD, further investigation is required. Worldwide, only about 300 individuals have been described so far with 179 distinct *ATP2C1* variants (Nellen et al. 2017). The majority of them are Asians, and only few cases from Central Europe were published, including a not genotyped case report from Poland (Rácz et al. 2005; Sudbrak et al. 2000; Chlebicka et al. 2012).

Herein, we report the results of the first genetic investigation in 18 Polish HHD patients together with characterization of splicing mutations and in silico structural dynamic modeling of novel missense mutations.

## Patients and methods

Eighteen probands of Polish descent (Table 1) with clinical HHD manifestation (according to Matsuda et al. (2014)) have been enrolled in the study, together with their relatives, if available. The average age at diagnosis was 29 years old (range: 15–40). All patients gave informed consent for participation.

**Table 1:** Results of genotyping

Fam. No.	Chr3(GRCh37):	HGVS ver.15.11 NM_014382.3:	HGVS ver.15.11 NP_055197.2:	Localization	Putative protein domain	No of probands (and family data)	Classification ACMG	Prediction algorithms	Additional data
Novel mutations									
1, 2	g.130656269 T > G	c.325-3 T > G	p.Ala109_Gln120del†	Intron 4	M2	2: detected in two distinct families (family 1: the first case in the family, the young-adult son of the patient have some slight clinical symptoms, but did not agree for clinical evaluation and genetic test, family 2 - no data)	Likely Pathogenic (PM4,PM2, PP3, PP4)	MaxEnt: − 100.0%	mRNA analysis performed
								NNSPLICE: − 99.1%	
								HSF: − 3.1%	
3	g.130660434G > T	c.423-1G > T	p.?	Intron 6	S2 or A	1 (DNA of affected son of the proband not analyzed)	Pathogenic (PVS1, PM2, PP3)	MaxEnt: − 100.0%	Canonical splice site
								NNSPLICE: − 100.0%	
								HSF: − 100.0%	
4	g.130678186G > A	c.899 + 1G > A	p.?	Intron 11	M4	1 (mutation present in proband and in his affected father)	Pathogenic (PVS1, PM2, PP3)	MaxEnt: − 100.0%	Canonical splice site
								NNSPLICE: − 100.0%	
								HSF: − 100.0%	
5	g.130698132C > G	c.1610C > G	p.Thr537Arg	Exon 18	N	1 (mutations in cis present in patient and his affected mother)	Likely Pathogenic (PM1, PM2, PP2, PP3)	PolyPhen-2: Probably damaging (score: Hum Div 0.985/1, Hum Var: 0.924/1)	In silico modeling performed
								SIFT (v6.2.0): Deleterious (score: 0.03, median: 3.58)	
								MutationTaster (v2013): disease causing (p value: 1)	
5	g.130698266A > G	c.1741 + 3A > G	p.Val524_Ile580del†	Intron 18	N		Likely Pathogenic (PM4,PM2, PP3, PP4)	MaxEnt: − 100.0%	mRNA analysis performed
								NNSPLICE: − 90.9%	
								HSF: − 23.2%	
6	g.130698138 T > C	c.1616 T > C	p.Leu539Pro	Exon 18	N	1 (no family data available)	Likely Pathogenic (PM1, PM2, PP2, PP3)	PolyPhen-2: Probably damaging (score: Hum Div 0.992/1, Hum Var: 0.936/1)	In silico modeling performed
								SIFT (v6.2.0): Tolerated (score: 0.05, median: 3.58)	
								MutationTaster (v2013): disease causing (*p* value: 1)	
7	g.130717154_130717166dup	c.2408_2420dup	p.Thr808TyrfsTer16	Exon 25	L4	1 (no family data available)	Pathogenic (PVS1, PM2, PP3)	PTC	The new reading frame ends in a STOP codon 16 positions downstream from Thr808
8,9,10,11	g.130718422G > T	c.2548G > T	p.Gly850Ter	Exon 26	M9	4 (in 3 cases mutations were present in at least two affected members of the family, in one case the DNA of several affected relatives of the proband were not analyzed	Pathogenic (PVS1, PM2, PP3)	PTC	The mRNA produced might be targeted for nonsense mediated decay (NMD)
12	g.130718438A > G	c.2564A > G	p.Gln855Arg	Exon 26	M9	1 (no family data available)	Likely Pathogenic (PM2, PP2, PP3)	PolyPhen2:Probably damaging (score: Hum Div 1/1, Hum Var: 1/1)	In silico modeling performed
								SIFT (v6.2.0): Deleterious (score: 0, median: 3.58)	
								MutationTaster (v2013): disease causing (p value: 1)	
Recurrent mutations							Reference		
13	g.130660532dupA	c.519dup	p.Arg174ThrfsTer4	Exon 7	A	1 (mutation present in proband and his child, in whom symptoms appear)	Dobson-Stone et al. 2002		
14	g. 130672792G > A	c.659G > A	p.Gly220Glu	Exon 8	A	1 (no familial data available)	Nellen et al. 2017		
15	g. 130,682,919 T > C	c.1004 T > C	p.Leu335Pro	Exon 12	S4	1 (mutation found in proband and 2 affected 1st degree relatives)	Ma et al. 2008		
16	g. 130698260A > G	c.1738A > G	p.Ile580Val	Exon 18	N	1 de novo (mutation not detected in parental DNA)	Dobson-Stone et al. 2002		
17	g. 130715627dupC	c.2234dup	p.Ala746SerfsTer11	Exon 23	M6	1 (no familial data available)	Meng et al. 2015		

**†**according to mRNA analysis

*A* Actuator domain; *N* nucleotide-binding domain; *S* (1–5) stalk helices in the cytoplasm; *M* (1–10) transmembrane helices

All coding exons of *ATP2C1* were analyzed using Sanger sequencing (primers and PCR conditions available on request) or panel next generation sequencing (customized KAPA Library Preparation Kit - Roche) using MiSeq (Illumina). The variants were annotated against NCBI RefSeq: NM_014382.3 and checked for presence in the GnomAD, ClinVar, HGMD Professional and *ATP2C1* LOVD v.3.0 databases.

Novel missense mutations were analyzed using in silico algorithms: DANN, MutationTaster, FATHMM, FATHMM-MKL, GERP, MutationAssessor, SIFT, Provean, and Poly-Phen2, classified according to ACMG guidelines (Richards et al. 2015) and visualized using dynamic structural modeling (Yasara Structure Package v.15.7.12). Briefly, isoform 1a of hSPCA1 (NP_055197.2) was modeled by homology, using eight closest templates identified in RCSB Protein Data Bank (PDB) records, IDs: 3N5K, 4BEW, 1WPG, 2YN9, 2YFY, 2ZXE, 4RET, and 4HYT. For each template, up to five alternative sequential alignments have been tested. Finally, the best scored models were built on the basis of 1WPG (37.2% of sequence identity and 56.7% of sequence similarity within 802 residues of 919 being aligned) and 3N5K (36.8%, 56.2%, and 810, respectively) PDB records. However, the final hybrid model, which combines the optimal parts of the top models, was scored substantially higher than the latter and thus was further used.

**Fig. 1:**
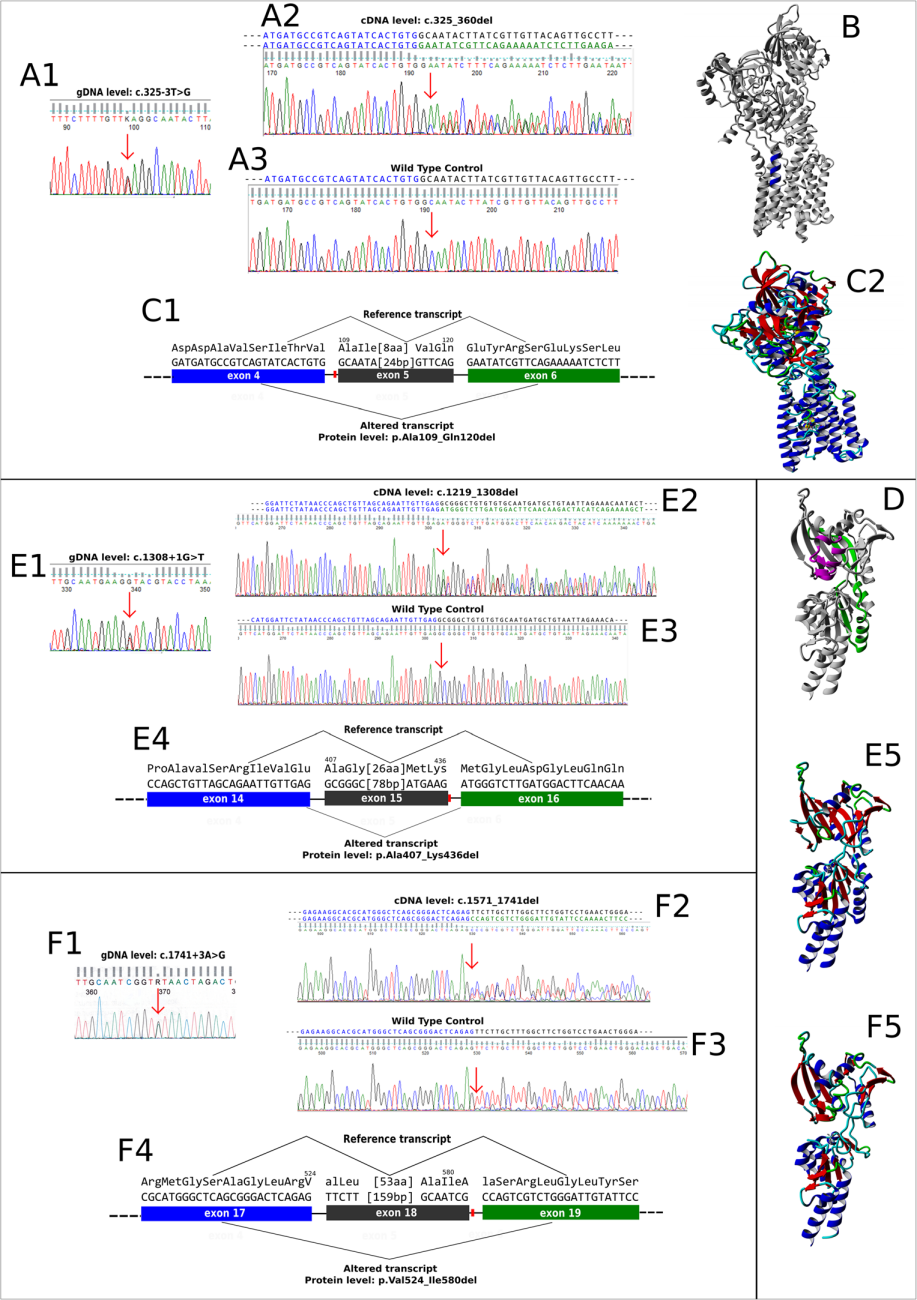
Results of functional analysis of three splicing mutations in *ATP2C1* gene and modeling of altered protein products (ATPase2C1). Results of DNA genotyping: A1, E1, F1, nucleotide substitutions indicated by red arrows; results of cDNA analysis: A2, E2, F2 (patients samples) and control samples (A3, E3, F3); schematic view of altered and normal transcripts (C1, E4, F4); predicted structure models of ATPase2C1 protein: B – wild type protein showing organization of transmembrane helices with amino acids 109–120 marked in blue; C2- protein lacking amino acids 109–120; D – wild type protein showing organization of ATP binding domain (amino acids 407–436 marked in magenta, amino acids 524–580 marked in green); E5 – protein lacking amino acids 407–436, F5 – protein lacking amino acids 524–580

Novel intronic mutations were evaluated with the use of three splice site prediction algorithms: MaxEnt, NNSPLICE, and HSF. In order to confirm the putative cryptic splicing of mutations c.325-3 T > G and c.1741 + 3A > G, RNA was isolated from peripheral blood leukocytes, reverse transcribed, and PCR amplified and analyzed using Sanger sequencing (Fig. 1). As negative and positive controls, we included RNA isolated from a healthy person and from a HHD patient with the already known mutation c.1308 + 1G > T.

## Results

*ATP2C1* variants were detected in 17/18 probands, resulting in a detection frequency of 94%. Overall, we detected 13 different heterozygous *ATP2C1* variants, (6 missense or nonsense, 3 splice site, 1 complex allele (missense and intronic *in cis*), and 3 deletions or duplications). Eight of them (8/13, 61%, Table 1) are novel, i.e., c.2548G > T, c.325-3 T > G, c.423-1G > T, c.899 + 1G > A, c.1616 T > C, c.2408_2420dup, c.2564A > G, and a complex allele: c.[1610C > G;1741 + 3A > G]. Identified mutations localize in the following exons: 26 (2/13), 18 (3/13), and (single variant each) in 7, 8, 12, 21, 23, and 25 and introns 4, 6, and 11.

The molecular dynamic modeling or/and in silico prediction analysis (Table 2) together with mRNA analysis of putative splicing mutations (Fig. 1) enabled us to confirm the likely pathogenic status of these novel variants. Precisely, novel c.325-3 T > G, c.1741 + 3A > G, and recurrent c.1308 + 1G > T mutations cause in-frame skipping of exons 5, 18, and 15, respectively, which seems to severely affect the protein structure.

**Table 2:**
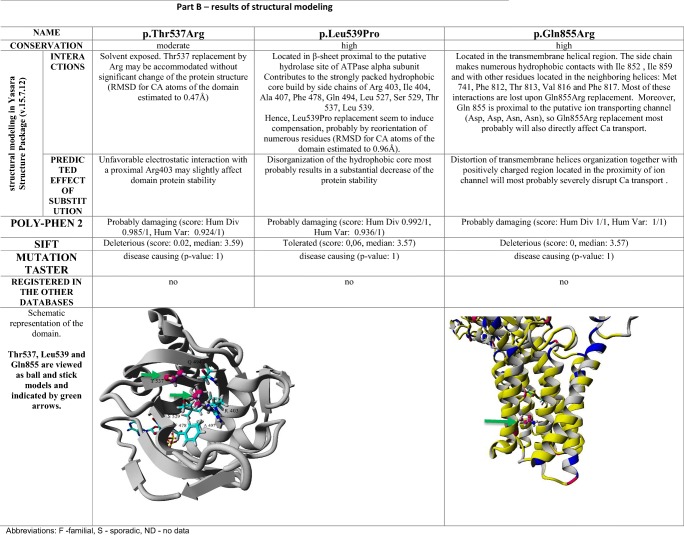
Results of structural dynamic modeling*.*

## Discussion

The majority of mutations (61%) identified in this study have never been reported before, including two recurrent novel splice site c.325-3 T > G (intron 4) and nonsense c.2548G > T (exon 26) mutations, identified in 2/17 (12%) and 4/17 (24%) in different Polish families, respectively. This could suggest specific founder mutations in this ethnic population. All *ATP2C1* missense mutations are localized in exons 8, 12, 18, and 26, which is partially in concordance with previous observations clustering in exons 12, 13, 18, 21 and 23 (Micaroni et al. 2016).

Half (4/8) of the novel mutations could easily be classified as pathogenic due to introduction of a premature stop codon (p.Gly850Ter, p.Thr808TyrfsTer16) or change in the conserved consensus sequence of the canonical splice sites (c.423-1G > T, c.899 + 1G > A). The novel missense mutations, p.Gln855Arg, p.Leu539Pro and the p.Thr537Arg detected *in cis* with 1741+3A>G, were analysed using molecular dynamic modeling and standard in silico tools.

Molecular dynamic modeling showed that conversion of Gln855 into Arg would distort the transmembrane helical structure and form a positively charged region located in the proximity of the ion channel, which seemingly would affect protein location and Ca^2+^ transport. The effect of p.Leu539Pro is less clear; however it is possible that this substitution would destabilize hydrophobic core formed between β-sheet structures and hence influence ATPase alpha subunit interactions, which in turn could affect ATP binding, its hydrolysis, and finally ion transportation. Unfortunately, no family data were available for probands with p.Leu539Pro and p.Gln855Arg; thus the genotype-phenotype segregation could not be performed.

The clinical significance of another missense, the p.Thr537Arg in exon 18 is more difficult to evaluate. In GnomAD, no records for p.Thr537Arg can be found. The prediction algorithms (PolyPhen, SIFT, MutationTaster) indicated possible pathogenic effect of p.Thr537Arg. Contradictory to them, dynamic structural modeling showed that this solvent exposed substitution most probably does not result in significant conformational change. Furthermore, the p.Thr537Arg was found *in cis* with a novel, mutation c.1741 + 3A > G in intron 18, which leads to exon 18 in-frame skipping as we have shown by mRNA analysis. Thus, the protein, if at all synthetized, lacks 57 codons including codon 537. This example of a complex allele containing two variants is not reported before, and c.[1610C > G;1741 + 3A > G], which both were assigned as potentially pathogenic by common prediction algorithms, draws attention on an important issue of careful pathogenicity status evaluation, especially when only selected exons are investigated. Importantly, when p.Thr537Arg status was evaluated alone, it was assigned as “likely pathogenic” using ACMG classification (Richards et al. 2015), which later changed into “uncertain significance” when we detected c.1741 + 3A > G and proved its impact on splicing.

Novel c.325-3 T > G and recurrent c.1308 + 1G > T mutations also lead to in-frame exons skipping (of exons 5 and 15, respectively). Moreover, given that skipping of exons 5 and 15 due to other mutations have been described before (Kitajima 2002; Matsuda et al. 2014; Xiao et al. 2019), our observation indicates that despite distinct molecular lesions, the functional effect of mutations may be similar, which could be significant with regard to the purposes of personalized treatment.

In summary, this is the first report of genetic analysis in Polish HHD patients. Thirteen variants were identified and characterized, including eight unreported before and two recurrent. The results further show heterogeneity in the *ATP2C1* mutational spectrum, with possible ethnic-specificity. Last but not the least, by showing a case of complex allele c.[1610C > G;1741 + 3A > G], we also point that careful in silico and extended molecular analysis is essential with respect to proper interpretation of mutation pathogenicity.
